# Fungal Isocyanide Synthases and Xanthocillin Biosynthesis in Aspergillus fumigatus

**DOI:** 10.1128/mBio.00785-18

**Published:** 2018-05-29

**Authors:** Fang Yun Lim, Tae Hyung Won, Nicholas Raffa, Joshua A. Baccile, Jen Wisecaver, Antonis Rokas, Frank C. Schroeder, Nancy P. Keller

**Affiliations:** a Department of Medical Microbiology and Immunology, University of Wisconsin—Madison, Madison, Wisconsin, USA; b Boyce Thompson Institute and Department of Chemistry and Chemical Biology, Cornell University, Ithaca, New York, USA; c Department of Biological Sciences, Vanderbilt University, Nashville, Tennessee, USA; d Department of Bacteriology, University of Wisconsin—Madison, Madison, Wisconsin, USA; Universidade de Sao Paulo

**Keywords:** Aspergillus fumigatus, isocyanide synthase, copper, melanocin, xanthocillin

## Abstract

Microbial secondary metabolites, including isocyanide moieties, have been extensively mined for their repertoire of bioactive properties. Although the first naturally occurring isocyanide (xanthocillin) was isolated from the fungus Penicillium notatum over half a century ago, the biosynthetic origins of fungal isocyanides remain unknown. Here we report the identification of a family of isocyanide synthases (ICSs) from the opportunistic human pathogen Aspergillus fumigatus. Comparative metabolomics of overexpression or knockout mutants of ICS candidate genes led to the discovery of a fungal biosynthetic gene cluster (BGC) that produces xanthocillin (*xan*). Detailed analysis of xanthocillin biosynthesis in A. fumigatus revealed several previously undescribed compounds produced by the *xan* BGC, including two novel members of the melanocin family of compounds. We found both the *xan* BGC and a second ICS-containing cluster, named the copper-responsive metabolite (*crm*) BGC, to be transcriptionally responsive to external copper levels and further demonstrated that production of metabolites from the *xan* BGC is increased during copper starvation. The *crm* BGC includes a novel type of fungus-specific ICS-nonribosomal peptide synthase (NRPS) hybrid enzyme, CrmA. This family of ICS-NRPS hybrid enzymes is highly enriched in fungal pathogens of humans, insects, and plants. Phylogenetic assessment of all ICSs spanning the tree of life shows not only high prevalence throughout the fungal kingdom but also distribution in species not previously known to harbor BGCs, indicating an untapped resource of fungal secondary metabolism.

## IMPORTANCE

Fungal ICSs are an untapped resource in fungal natural product research. Their isocyanide products have been implicated in plant and insect pathogenesis due to their ability to coordinate transition metals and disable host metalloenzymes. The discovery of a novel isocyanide-producing family of hybrid ICS-NRPS enzymes enriched in medically and agriculturally important fungal pathogens may reveal mechanisms underlying pathogenicity and afford opportunities to discover additional families of isocyanides. Furthermore, the identification of noncanonical ICS BGCs will enable refinement of BGC prediction algorithms to expand on the secondary metabolic potential of fungal and bacterial species. The identification of genes related to ICS BGCs in fungal species not previously known for secondary metabolite-producing capabilities (e.g., *Saccharomyces* spp.) contributes to our understanding of the evolution of BGC in fungi.

## INTRODUCTION

Microbial natural products or specialized metabolites have been extensively mined for their pharmaceutical potential since the serendipitous discovery of penicillin by Alexander Fleming in 1928 that brought about the Golden Age of antibiotics. Unfortunately, years of mining our soils for new antibiotics have seen a rapid exhaustion of this resource pool, leading to unacceptably high rediscovery rates of the same class of compounds. Coupled with the concerning rise of antibiotic resistance and dwindling options for new effective antibiotics, the field of drug discovery is being ushered into finding alternative ways to mine new and unique microbial compounds. Today, our deeper understanding of the pathways and specialized enzymes involved in microbial secondary metabolite biosynthesis has enabled us not only to mine the wealth of sequenced microbial genomes for novel and unique bioactive natural products but also to generate methods for a sustainable source of these compounds.

**FIG 1:**
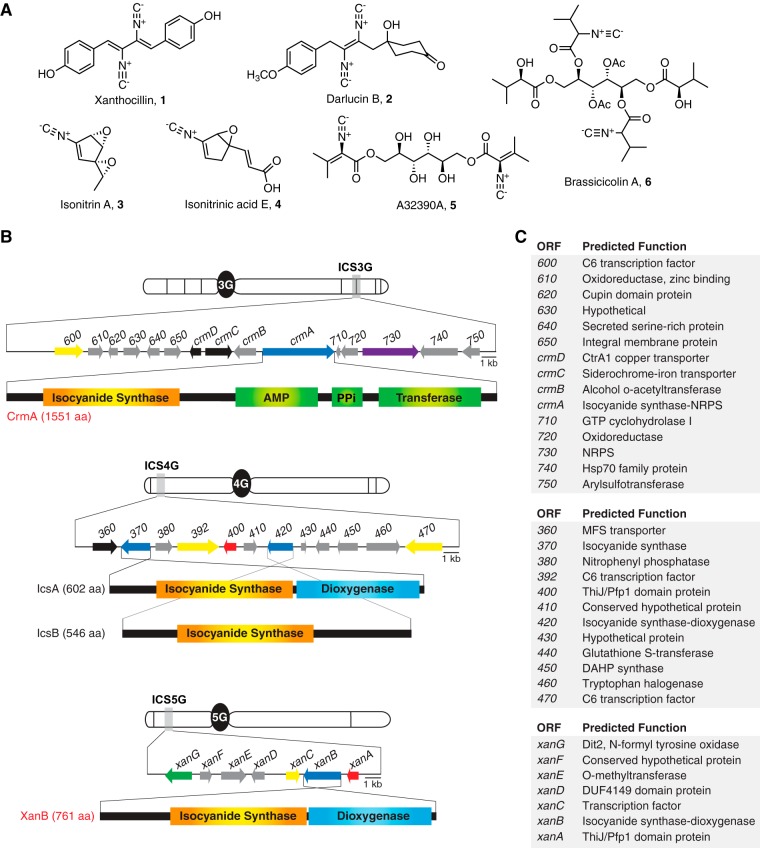
Isocyanides identified from fungi and architecture of isocyanide synthase-containing gene clusters in A. fumigatus. (A) Examples of isocyanides identified from fungi, including xanthocillin (compound 1), darlucin B (compound 2), isonitrin A (compound 3), isonitrinic acid E (compound 4), A32390A (compound 5), and brassicicolin A (compound 6). (B) The *crm* gene cluster contains a novel multidomain ICS-NRPS hybrid enzyme. The 4G gene cluster contains two ICS domain genes, *icsA* and *icsB*. The *xan* gene cluster contains an ICS domain gene, *xanB*. Isocyanide synthases are depicted in blue. Fungal C6 transcription factors are depicted in yellow. Transporters are depicted in black. ThiJ/Pfp1 domain proteins with homology to isocyanide hydratases are depicted in red. P450 monooxygenases are depicted in green. (C) Predicted function of open reading frames (ORFs) within A. fumigatus isocyanide gene clusters.

Isocyanides (or isonitriles) are a class of microbial secondary metabolites long pursued for their broad repertoire of pharmacological properties. Since the isolation of the first naturally occurring isocyanide, xanthocillin (compound 1), from Penicillium notatum in 1950 (1), diverse isocyanide-containing natural products have been isolated from marine and terrestrial sources, including marine sponges (2, 3), nudibranch molluscs (4), bacteria (5–8), and fungi (9–11) (Fig. 1A). Many marine isocyanides are nitrogenous terpenes known as isocyanoterpenes, where the terpenoid carbon scaffolds are adorned with various isocyanide, formamide, isocyanate, or isothiocyanate functional groups (3). Isocyanides from terrestrial sources, on the other hand, are mostly derived through modification of α-amino acids or indole alkaloids, which in some cases are glycosylated (5). The presence of an isonitrile moiety imparts unique biological (e.g., cytotoxic, antibacterial, and antiprotozoal) and chemical (e.g., transition metal coordination) properties and has enabled synthetic and biochemical (e.g., “click” chemistry) applications (12, 13). In addition to industrial and pharmaceutical applications, naturally occurring bacterial isocyanides such as the diisonitrile SF2768 from Streptomyces thioluteus (7) have been shown to function as chalkophores: natural products involved in copper chelation and extracellular copper uptake. Due to their ability to form coordination complexes with various transition metals, there is growing evidence that isocyanides play an important role in pathogenesis of insect, plant, and human diseases (5, 8, 10).

Despite their broad range of applications and emerging impact on various host-pathogen interactions, relatively little is known about the biosynthesis or molecular switches governing the production of isocyanides, in contrast to the well-studied nonribosomal peptide and polyketide biosynthetic pathways. The first insights into microbial isocyanide biosynthesis were enabled by the discovery of bacterial synthases termed isonitrile synthases (ISNs) or isocyanide synthases (ICSs) (e.g., PvcA [6, 14], IsnA [5, 15], AmbI1/AmbI2 [16], and WelI1/WelI2 [16]) shown to be responsible for the conversion of amino groups into isonitrile moieties in aromatic amino acids (15, 17). More recently, two new biosynthetic routes to bacterial isocyanide production have been reported: one that requires the activity of two modifying enzymes, a thioesterase homologue (ScoD) and a nonheme iron(II)-dependent oxidase (ScoE), identified in Mycobacterium tuberculosis to produce isonitrile lipopeptides (8), in addition to another that requires the activity of a typical nonribosomal peptide synthetase (SfaD), identified in S. thioluteus to produce the diisonitrile SF2768 (7).

Although fungi are known producers of various isocyanide compounds, including xanthocillin, the first isocyanide to be isolated, the biosynthetic origins of fungal isocyanides remain unknown. Production of xanthocillin-like isocyanides such as BU-4704 (18) and xanthoascin, an isocyanide first isolated from Aspergillus candidus (9, 19), have been reported from Aspergillus fumigatus, a filamentous mold that is both a ubiquitous compost-loving carbon-nitrogen recycler and a highly fatal opportunistic pathogen of immunocompromised individuals (20). Given the emerging role of isocyanide compounds during pathogenesis of insect (5), plant (5, 10), and human (8) pathogens, we sought to identify the biosynthetic origins of fungal isocyanides in this opportunistic pathogen.

Here we present the first fungal isocyanide biosynthetic pathway responsible for production of xanthocillin-like isocyanides and two previously undescribed melanocin-like dipeptides in A. fumigatus. Via genome mining, we identified four isocyanide synthases (ICSs) parsed across three biosynthetic gene clusters (BGCs) (Fig. 1B) within the A. fumigatus genome: notably, three of the four ICSs reside in BGCs undetected by current *in silico* BGC prediction algorithms. Given the cryptic nature of these BGCs in A. fumigatus, we expanded our search for ICSs and found widespread occurrence of ICS homologues within the fungal kingdom, including distribution within fungal taxa not generally recognized to harbor secondary metabolite-producing BGCs. An exhaustive search for ICS-containing genes spanning the tree of life identified three ICS enzyme variations: homologues of monodomain isocyanide synthases (ICSs), putative two-domain isocyanide synthase-dioxygenases (ICS-DOX), and a novel group of multidomain putative isocyanide synthase-nonribosomal peptide synthases (ICS-NRPSs), which are found predominantly in fungal pathogens. We further demonstrate the production of isocyanides and transcription of their biosynthetic enzymes in A. fumigatus to be copper responsive, and we show that ICS overexpression induces cellular copper starvation.

## RESULTS

### Isocyanide synthase biosynthetic gene clusters in Aspergillus fumigatus.

Amino acid sequences of two bacterial ICSs (PvcA [6] and IsnA [15]) were queried against the A. fumigatus AF293 genome using PSI-BLAST (21). Four ICS domain proteins were identified: AFUA_3G13690/AFUA_3G13700 (CrmA, found to be transcribed as a single mRNA encoded by both AFUA_3G13690 and AFUA_3G13700 [see Fig. S1 in the supplemental material]), AFUA_4G01370 (IcsA), AFUA_4G01420 (IcsB), and AFUA_5G02660 (XanB) (Fig. 1B). Published transcriptomic data of A. fumigatus indicate that these putative ICSs are located within three distinct BGCs (ICS3G, ICS4G, and ICS5G), based on evidence for coregulation of their flanking genes (22–24). CrmA, a novel fungus-specific multidomain ICS-nonribosomal peptide synthetase (ICS-NRPS), was predicted by available BGC prediction algorithms (25, 26) to be part of a 15-gene NRPS BGC. Notably, neither the *icsA*/*icsB*- nor the *xanB*-containing cluster (ICS4G and ICS5G, respectively) was detected by BGC prediction algorithms, likely due to the absence of canonical secondary metabolite-producing synthases (i.e., polyketide synthases, NRPSs, terpene cyclases, dimethylallyl tryptophan synthases, or fatty acid synthases) within these gene clusters. Multiple-sequence alignment comparing all four A. fumigatus ICS amino acid sequences to that of biochemically confirmed bacterial ICSs (PvcA and IsnA) revealed conservation of key amino acid residues presumed to be important for the function of this family of enzymes (see Fig. S2 in the supplemental material) (14).

10.1128/mBio.00785-18.1 FIG S1  Gene configuration of *crmA*. (A) Current prediction indicated that *crmA* (AFUA_3G13690) is a 1,742-bp gene with three introns. (B) New AUGUSTUS prediction indicated that *crmA* is a 5,082-bp gene that spans both AFUA_3G13690 and AFUA_3G13700 and contains seven introns. An additional intron (denoted by a navy blue bar) is predicted to span the 3′ end of AFUA_3G13690 extending into the intergenic region between AFUA_3G13690 and AFUA_3G13700. (C) PCR amplification of intron (marked by an asterisk in fragment 1) and fusion (fragment 2) regions using cDNA synthesized from total RNA isolated under conditions with inducing YG medium (lanes 1 and 5) and noninducing YG medium plus trace elements (lanes 2 and 6). The negative no-template control is shown in lanes 3 and 7, and the positive genomic DNA control is shown in lanes 4 and 8. A white arrow marks the presence of the transcribed fusion region from cDNA obtained specifically under the inducing condition. (D) *crmA* sequence obtained from cDNA synthesized from total RNA isolated under the inducing YG medium condition and cloned into a bacterial expression vector, pTEV5 (48). The sequences shaded in yellow denote current prediction of *crmA* (AFUA_3G13690). Sequences shaded in red denote current prediction of AFUA_3G13700. Regions shaded in blue denote introns. Both ALLseq and pTEV-dit1nrps denote sequences obtained from cDNA synthesized from total RNA extracted from the inducing condition and cloned into pTEV5. dit1-NRPS gDNA denotes the sequence of the gDNA region that spans AFUA_3G13690 to AFUA_3G13700. “Majority” denotes the consensus sequence of *crmA* upon alignment of all three DNA sequences above using MegAlign within the Lasergene13 suite (DNASTAR, Madison, WI). Download FIG S1, EPS file, 15.9 MB. Copyright © 2018 Lim et al. 2018 Lim et al. This content is distributed under the terms of the Creative Commons Attribution 4.0 International license.

10.1128/mBio.00785-18.2 FIG S2  Multiple-sequence alignment of fungal isocyanide synthases with PvcA and IsnA. The A. fumigatus ICSs are highlighted in red. The amino acid residues of six conserved motifs based on the crystal structure of PvcA are highlighted in individual black boxes along with their corresponding motif numbers. Red asterisks mark amino acid residues proposed to be important in the phosphate binding pocket of PvcA based on 3D modeling of the crystallized protein structure. AN2705 is one of the two predicted ICSs in A. nidulans. AFLA_058950 and AFLA_102560 are two ICSs found in A. flavus. ACLA_064260, ACLA_061750, and ACLA_102560 are three ICSs found in A. clavatus. Download FIG S2, EPS file, 2.6 MB. Copyright © 2018 Lim et al. 2018 Lim et al. This content is distributed under the terms of the Creative Commons Attribution 4.0 International license.

### Expansion of ICS domain proteins across the tree of life.

**FIG 2:**
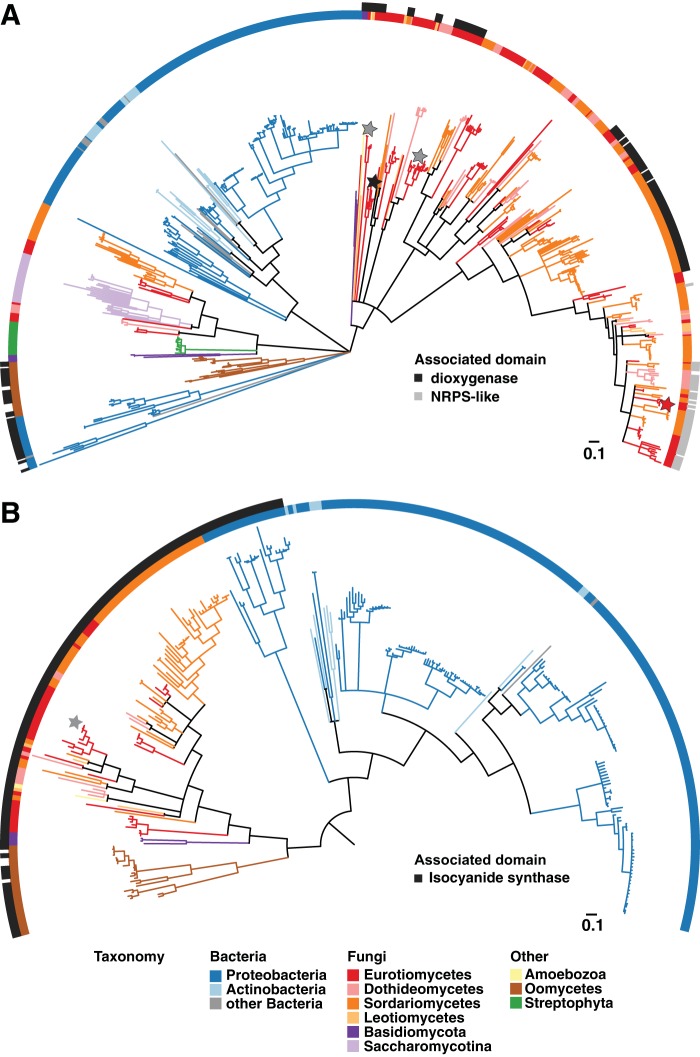
Evolutionary relationship of isocyanide synthase and dioxygenase domain proteins across the tree of life. (A) Maximum likelihood phylogeny of the isocyanide synthase (PF05141) domain proteins. Color strips along the tree perimeter correspond to taxonomy (inner strip) and the presence of additional Pfam domains (outer strip): taurine catabolism dioxygenase, PF02668 (black); and NRPS-like, PF00501 and PF02458 (gray). The four A. fumigatus AF293 proteins in the tree are starred: one protein consists of just the isocyanide synthase (PF05141) domain (black star), two proteins contain an additional dioxygenase (PF02668) domain (gray stars), and one protein contains additional NRPS (PF00501 and PF02458) domains (red star). (B) Maximum likelihood (53) phylogeny of taurine catabolism dioxygenase (PF02668) domain proteins. Color strips along the tree perimeter correspond to taxonomy (inner strip) and the presence of an isocyanide synthase domain, PF05141 (black). The two A. fumigatus AF293 proteins in the tree are starred (gray); both contain a PF05141 domain. The trees were midpoint rooted and visualized using iTOL version 3.0. Branches with bootstrap support of less than 50 were collapsed using TREECOLLAPSERCL4 version 4.0 (http://emmahodcroft.com/TreeCollapseCL.html).

Phylogenetic analysis of the ICS domain (PF05141)-containing proteins (from here on referred to as ICS proteins) showed that all four A. fumigatus Af293 ICS proteins group within the fungal clades (Fig. 2A; see Fig. S3 in the supplemental material). The taxonomic diversity and tree topology of PF05141 suggest this domain has a complex evolutionary history, including multiple gene duplication, loss, and horizontal transfer events; however, the exact timing and locations of these events are unclear. In bacteria, ICS proteins are distributed mainly across *Proteobacteria*, *Cyanobacteria*, and *Actinobacteria* (Fig. 2A and Fig. S3). In eukaryotes, ICS proteins are widespread across the fungal kingdom but are found only sporadically in 10 species of oomycetes, one species of Amoebozoa, and one species of moss (Fig. 2A and Fig. S3).

10.1128/mBio.00785-18.3 FIG S3  Maximum likelihood phylogeny of isocyanide synthase (PF05141) domain proteins across the tree of life. The phylogeny was arbitrarily rooted near the midpoint and visualized using iTOL version 3.0 (53). Branches with bootstrap support of less than 50 were collapsed using TREECOLLAPSERCL4 version 4.0 (http://emmahodcroft.com/TreeCollapseCL.html). Numbers below branches indicate bootstrap support. The color strips to the right correspond to the taxonomic lineages and presence of additional Pfam domains (see legend). Sequence names have the following structure: ensemble protein id-start stop (alignment region)-NCBI taxonomy id-species name (truncated, 20 characters maximum)-lineage. The four A. fumigatus Af293 proteins are highlighted in yellow. The sequence alignment and tree file used to draw this phylogeny are available for download on the figshare data repository (https://doi.org/10.6084/m9.figshare.4721116.v1). Download FIG S3, PDF file, 0.2 MB. Copyright © 2018 Lim et al. 2018 Lim et al. This content is distributed under the terms of the Creative Commons Attribution 4.0 International license.

Within the kingdom Fungi, ICS proteins are highly prevalent in the phylum Ascomycota; in addition, they are also found in four species of the phylum Basidiomycota (Fig. 2A; see Fig. S3 and Table S1 in the supplemental material). In the Ascomycota, we find ICS proteins to be most common throughout the Pezizomycotina (Fig. 2A and Fig. S3); however, they also occur in 20 species of Saccharomycotina, including several clinically important *Candida* species and in the economically important baker’s yeast, Saccharomyces cerevisiae (Fig. 2A and Table S1). In these latter fungi, the ICS-homologous protein is known as Dit1, which has been shown to participate in the biosynthesis of the dipeptide dityrosine, an ascospore wall component (see below) (27).

10.1128/mBio.00785-18.7 TABLE S1  List of sequences with significant hits to the PF05141, PF02668, and PF00501 domains. Download TABLE S1, XLSX file, 0.3 MB. Copyright © 2018 Lim et al. 2018 Lim et al. This content is distributed under the terms of the Creative Commons Attribution 4.0 International license.

Our analysis revealed that ICS proteins commonly exhibit two distinct protein domain architectures in bacteria but three in eukaryotes: (i) single-domain ICSs found in both bacteria (e.g., P. aeruginosa PvcA) and eukaryotes (e.g., A. fumigatus IcsA and yeast Dit1), (ii) two-domain isocyanide synthase-dioxygenases (ICS-DOXs) found in both bacteria (e.g., V. cholerae IsnAB) and eukaryotes (e.g., A. fumigatus IcsB and XanB), and (iii) the novel multidomain isocyanide synthase-NRPSs (ICS-NRPSs) found only in fungi (e.g., A. fumigatus CrmA). To further map the distribution of DOX and NRPS-like domains that accompany these ICSs, we reconstructed the phylogeny of dioxygenase domain (PF02668)-containing proteins (Fig. 2B; see Fig. S4 in the supplemental material) and examined the taxonomic distribution of the NRPS-like (PF00501) domain-containing proteins (Table S1). Unlike bacterial PF02668-type dioxygenases, which most often exist without an accompanying ICS domain, it is notable that all fungal PF02668-type dioxygenase domain proteins exist as hybrid enzymes with ICSs (Fig. 2B and Fig. S4). Closer examination of the fungal species that harbor ICS-NRPS proteins showed a striking enrichment of fungal pathogens of humans, insects, and plants (Table S1).

10.1128/mBio.00785-18.4 FIG S4  Maximum likelihood phylogeny of the taurine catabolism dioxygenase (PF02668) domain proteins across the tree of life. The phylogeny was midpoint rooted and visualized using iTOL version 3.0 (53). Branches with bootstrap support of less than 50 were collapsed using TREECOLLAPSERCL4 version 4.0 (http://emmahodcroft.com/TreeCollapseCL.html). Numbers below branches indicate bootstrap support. The color strips to the right correspond to the taxonomic lineages and the presence of a pyoverdine/dityrosine biosynthesis domain, PF05141 (see legend). Sequence names have the following structure: ensemble protein id-start stop (alignment region)-NCBI taxonomy id-species name (truncated, 20 characters maximum)-lineage. The two A. fumigatus Af293 proteins containing the dioxygenase (PF02668) domain are highlighted in yellow. The sequence alignment and tree file used to draw this phylogeny are available for download on the figshare data repository (https://doi.org/10.6084/m9.figshare.4721116.v1). Download FIG S4, PDF file, 0.1 MB. Copyright © 2018 Lim et al. 2018 Lim et al. This content is distributed under the terms of the Creative Commons Attribution 4.0 International license.

### Uncovering fungal copper-responsive ICS gene clusters.

Isocyanides have long been recognized for their ability to form complexes with various transition metals (28). However, the effects of extra- and intracellular transition metal availability on the production of these compounds have not been explored. In a preliminary experiment to assess impact of metals on the expression of the four ICSs we identified in A. fumigatus, we found that several genes within the ICS3G BGC were expressed highly on undefined yeast extract-glucose (YG) medium but not on the standard *Aspergillus* defined glucose minimal medium (GMM) that is supplemented with transition metals, including zinc, manganese, iron, cobalt, copper, and molybdenum (data not shown).

**FIG 3:**
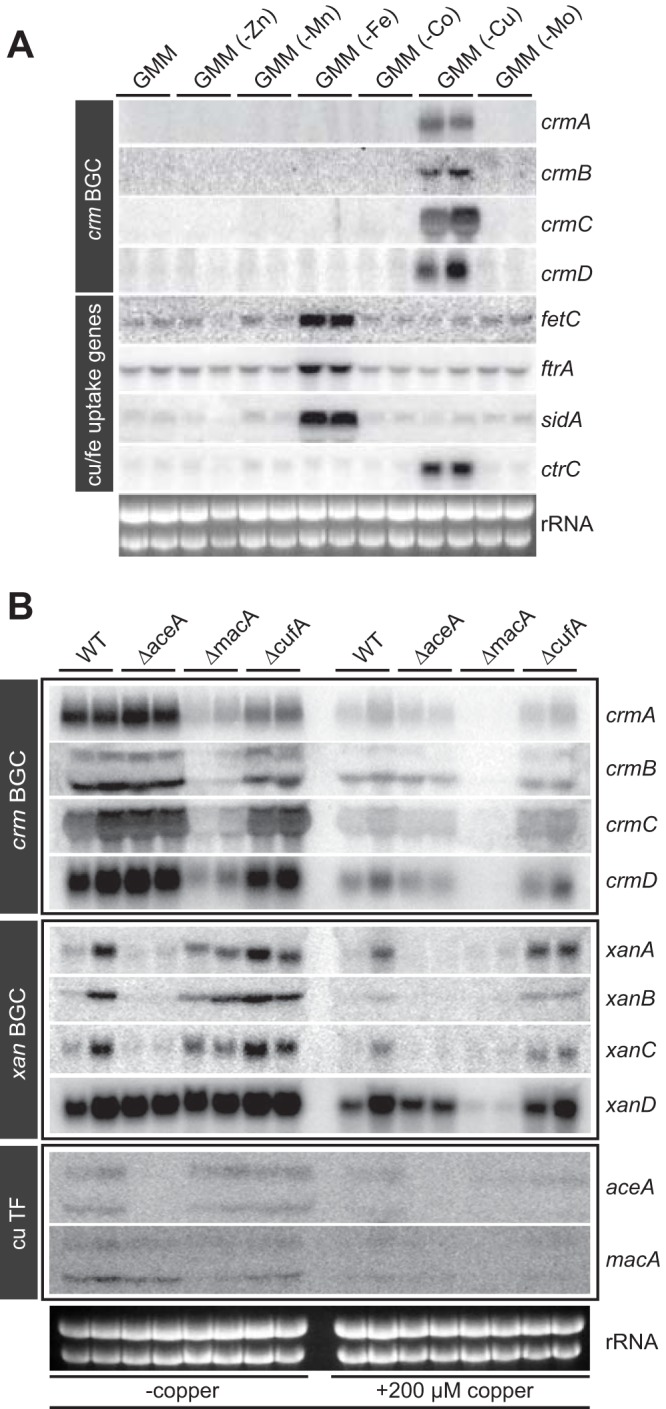
Copper-responsive expression of the *crm* gene cluster. (A) Northern analysis showing mRNA expression of *crmA* to -*D* of the wild-type fungus grown on GMM and GMM deprived of various components of the trace elements. Expression of known iron (*fetC*, *ftrA*, and *sidA*)- and copper (*ctrC*)-responsive genes was assessed. (B) Northern analysis showing mRNA expression of *crmA* to -*D* and *xanA* to -*D* in the copper-fist transcription factor mutant Δ*aceA*, Δ*macA*, and Δ*cufA* strains under copper-depleted conditions and 1 h posttreatment with 200 µM copper sulfate. Expression of both *macA* and *aceA* was also assessed under these conditions.

To further investigate the role of transition metal availability for ICS3G expression, the wild-type (WT) strain of A. fumigatus (AF293) was grown on a series of GMM formulations, each lacking one transition metal. We found that a subset of four genes (*crmA* to *crmD*) within the ICS3G BGC are expressed when copper is not supplemented (copper depleted) and refer to these four genes as the *crm* cluster (Fig. 3A). Two *crm* genes are predicted to encode copper (*crmD*) and iron (*crmC*) transporters (Fig. 1). To investigate the specificity of this transcriptional response, levels of expression of several verified copper- and iron-responsive genes were assessed. We found that expression patterns of the high-affinity copper transporter (*ctrC*) closely mimicked that of *crmA* to *crmD* (Fig. 3A). As expected, transcripts pertaining to high-affinity reductive iron uptake systems were upregulated in iron-depleted medium, whereas expression of *crmA* to *crmD* was not induced (Fig. 3A), demonstrating the specificity of the *crmA* to *-D* transcriptional response to copper.

To elucidate the regulatory network governing this copper-responsive transcriptional response, expression levels of *crmA* to *-D* were assessed in three copper-fist transcription factor mutants recently identified in A. fumigatus (29–31). MacA is important during copper starvation and induces a transcriptional network for copper uptake, AceA is crucial for initiating the detoxification program at toxic copper levels, and a CufA homologue plays a dual role in Cryptococcus neoformans (32), but its specific role or roles in A. fumigatus remain unclear. When grown in media lacking copper supplementation, we found that expression of *crmA* to -*D* is significantly reduced but not completely abolished in the Δ*macA* mutant (Fig. 3B), suggesting a crucial role by MacA for inducing expression of *crmA* to -*D* during copper starvation. MacA also was critical for *crm* gene expression under copper-replete conditions, whereas loss of *aceA* or *cufA* had no to minimal impact on *crm* transcription.

We also found that a subset of the *xan* genes were regulated by both AceA and MacA. In contrast to *crmA* to -*D*, our data indicate that AceA is required for the expression of *xanA* to *-C* during copper starvation and both AceA and MacA are important for *xan* gene expression under copper-replete conditions (Fig. 3B). CufA loss did not impact *xan* gene expression. Finally, we did not observe expression of the two remaining ICS genes, *icsA* and *icsB*, under any of the tested conditions (data not shown). Taken together, our results demonstrate that both AceA and MacA play distinct roles upon governance of two ICS gene clusters, *crm* and *xan*, under both copper-depleted and copper-replete conditions.

### The *xan* BGC encodes the xanthocillin biosynthetic pathway.

**FIG 4:**
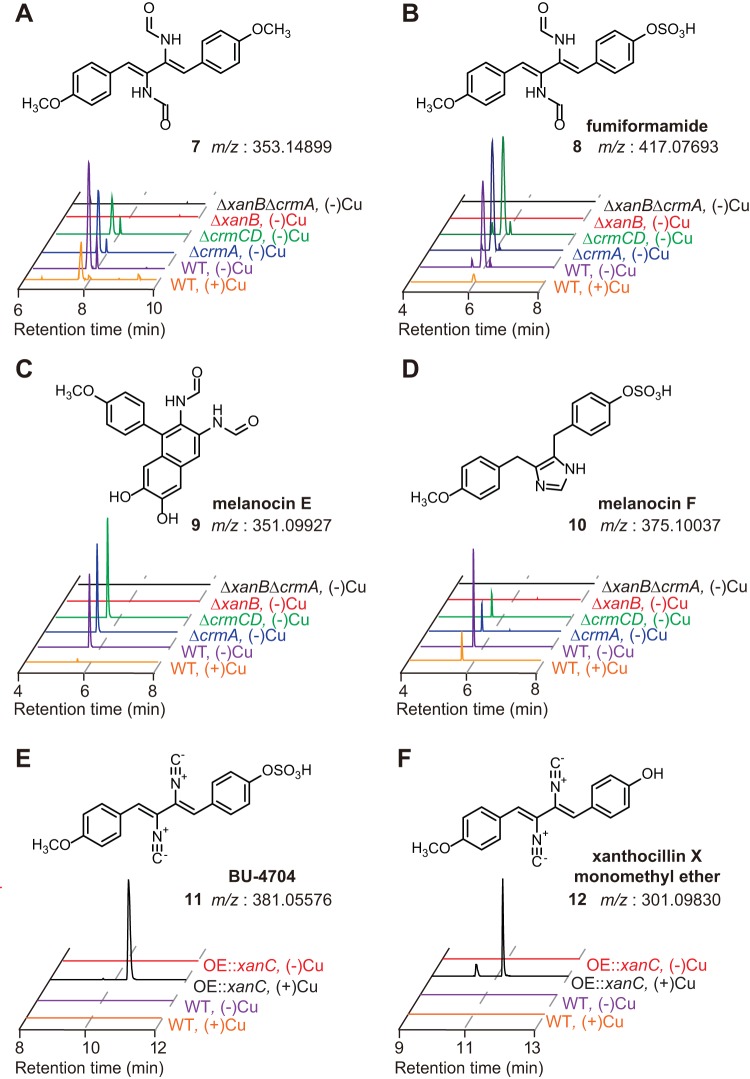
HRMS-based comparative metabolomics of AF293 (wild type [WT]) and A. fumigatus ICS mutants. (A to F) Extracted-ion chromatograms (EICs) corresponding to compounds 7 to 12 in the wild type grown with or without copper (orange and purple lines, respectively), in the Δ*xanB*, Δ*crmA*, and Δ*crmCD* mutants (blue, green, and red lines, respectively), the Δ*xanB* Δ*crmA* double mutant under the copper-depleted condition (black lines), and the OE::*xanC* mutant with or without copper (black and red lines, respectively).

Given that a previous study reported isolation of xanthocillin-like isocyanides from A. fumigatus (9), we asked whether any of the identified ICSs are involved in their biosynthesis. We constructed single and double ICS deletion mutants in the two BGCs expressed under our conditions: ICS3G (*crmA*) and ICS5G (*xanB*) (see Table S3 and Fig. S5 in the supplemental material). Given the transcriptional response to copper of *crmA* and *xanB* (Fig. 3), wild-type and deletion mutant strains were grown under both copper-depleted and copper-replete conditions. Metabolite extracts from the wild type and the three ICS deletion mutants were analyzed by liquid chromatography–high-resolution mass spectrometry (LC-HRMS), and the resulting metabolite profiles were compared using the XCMS data processing platform (33) (Fig. 4A to D).

10.1128/mBio.00785-18.5 FIG S5  Southern analysis of the Δ*crmA*, Δ*xanB*, *ΔxanB ΔcrmA*, *ΔcrmC ΔcrmD*, and OE::*xanC* mutants. (A) Restriction digest diagram of wild-type and mutant alleles. Radioactive probes used for Southern analysis are depicted in blue. (B) Southern blot of deletion mutants. “P” denotes the parental strain bearing the wild-type allele. Double black asterisks denote correct single integration of the mutant allele, and double red asterisks denote the specific mutant used in this study. Numbers correspond to the base pair length of each fragment Download FIG S5, EPS file, 9.1 MB. Copyright © 2018 Lim et al. 2018 Lim et al. This content is distributed under the terms of the Creative Commons Attribution 4.0 International license.

Comparing the *ΔcrmA* Δ*xanB* double mutant with the A. fumigatus wild type revealed four compounds (Fig. 4A to D) whose production was comparable to that of the wild type in the *ΔcrmA* mutant but undetectable in both the *ΔxanB* and *ΔcrmA* Δ*xanB* mutants. Analysis of their tandem mass spectrometry (MS/MS) spectra and molecular formulas suggested that these compounds represent xanthocillin derivatives. Isolation via preparative high-performance liquid chromatography (HPLC) followed by two-dimensional nuclear magnetic resonance (2D NMR) spectroscopic characterization revealed the two known metabolites, a dimethoxyl formyl xanthocillin derivative, *N*,*N*′-((1*Z*,3*Z*)-1,4-bis(4-methoxyphenyl)buta-1,3-diene-2,3-diyl)diformamide (compound 7) (Fig. 4A and Table 1; see Fig. S6A and Table S2A in the supplemental material), and the sulfated formyl xanthocillin derivative fumiformamide (compound 8) (Fig. 4B and Table 1) (9), which had previously been reported from A. fumigatus (9). Additionally we identified two novel compounds, a 1,2-diformylamido derivative we named melanocin E (compound 9) (Fig. 4C, Table 1, Fig. S6B, and Table S2B) due to its similarity to the previously described *Penicillium* (previously *Eupenicillium*) *shearii* metabolite melanocin C (34), and an imidazole-containing compound we named melanocin F (compound 10) (Fig. 4D, Table 1, Fig. S6C, and Table S2C), which is likely derived from reductive cyclization of compound 8 and similar to a synthetic compound previously obtained from cyclization of xanthocillin dimethyl ether with sodium dichromate (35).

10.1128/mBio.00785-18.6 FIG S6  NMR spectra of (A) *N*,*N*′-((1*Z*,3*Z*)-1,4-bis(4-methoxyphenyl)buta-1,3-diene-2,3-diyl)diformamide (compound 7), (B) melanocin E (compound 9), (C) melanocin F (compound 10), and (D) BU-4704 (compound 11). (i) ^1^H NMR, (ii) DQF-COSY, and (iii) coupled HSQC and (iv) HMBC spectra of compounds 7 and 9 to 11. Download FIG S6, EPS file, 6 MB. Copyright © 2018 Lim et al. 2018 Lim et al. This content is distributed under the terms of the Creative Commons Attribution 4.0 International license.

**TABLE 1:** UHPLC-HRMS data for compounds 7 to 12 in OE::*xanC* extracts

Compound	Observed *m*/*z*	Ion	Calculated ion formula	Calculated *m*/*z*	Retention time (min)	Yield of compound (mg/liter of culture)^a^
7	353.14899	[M + H]^+^	C_20_H_21_O_4_N_2_^+^	353.14958	7.69	~1–5
8	417.07693	[M − H]^−^	C_19_H_17_O_7_N_2_S^−^	417.07620	5.88	
9	351.09927	[M − H]^−^	C_19_H_15_O_5_N_2_^−^	351.09865	5.54	~1–5
10	375.10037	[M + H]^+^	C_18_H_19_O_5_N_2_S^+^	375.10092	5.63	~1–5
11	381.05576	[M − H]^−^	C_19_H_13_O_5_N_2_S^−^	381.05507	10.31	~0.5–1
12	301.09830	[M − H]^−^	C_19_H_13_O_2_N_2_^−^	301.09825	11.09	

a The numbers indicate estimated production of each compound in freshly prepared extracts. Compounds were isolated from OE::*xanC* mutant extracts.

10.1128/mBio.00785-18.8 TABLE S2  (A) ^1^H (800 MHz) and ^13^C (201 MHz) NMR spectroscopic data for *N*,*N*′-((1*Z*,3*Z*)-1,4-bis(4-methoxyphenyl)buta-1,3-diene-2,3-diyl)diformamide (compound 7) in dimethyl sulfoxide (DMSO)-*d*_6_. Chemical shifts were referenced to δ(CHD_2_SOCD_3_) = 2.50 and δ(^13^CHD_2_SOCD_3_) = 39.5. ^13^C chemical shifts were determined via HMBC and HSQC spectra. ^1^H, ^1^H-*J*-coupling constants were determined from the acquired ^1^H or DQF-COSY spectra. HMBC correlations are from the proton(s) stated to the indicated ^13^C atom. (B) ^1^H (800 MHz) and ^13^C (201 MHz) NMR spectroscopic data for melanocin E (compound 9) in methanol-*d*_4_. Chemical shifts were referenced to δ(CHD_2_OD) = 3.31 and δ(^13^CHD_2_OD) = 49.0. ^13^C chemical shifts were determined via HMBC and HSQC spectra. ^1^H, ^1^H-*J*-coupling constants were determined from the acquired ^1^H or DQF-COSY spectra. HMBC correlations are from the proton(s) stated to the indicated ^13^C atom. (C) ^1^H (800 MHz) and ^13^C (201 MHz) NMR spectroscopic data for melanocin F (compound 10) in methanol-*d*_4_. Chemical shifts were referenced to δ(CHD_2_OD) = 3.31 and δ(^13^CHD_2_OD) = 49.0. ^13^C chemical shifts were determined via HMBC and HSQC spectra. ^1^H, ^1^H-*J*-coupling constants were determined from the acquired ^1^H or DQF-COSY spectra. HMBC correlations are from the proton(s) stated to the indicated ^13^C atom. (D) ^1^H (800 MHz) and ^13^C (201 MHz) NMR spectroscopic data for BU-4704 (compound 11) in methanol-*d*_4_. Chemical shifts were referenced to δ(CHD_2_OD) = 3.31 and δ(^13^CHD_2_OD) = 49.0. ^13^C chemical shifts were determined via HMBC and HSQC spectra. ^1^H, ^1^H-*J*-coupling constants were determined from the acquired ^1^H or DQF-COSY spectra. HMBC correlations are from the proton(s) stated to the indicated ^13^C atom. Download TABLE S2, DOCX file, 0.2 MB. Copyright © 2018 Lim et al. 2018 Lim et al. This content is distributed under the terms of the Creative Commons Attribution 4.0 International license.

We found that all four xanthocillin derivatives were abundantly produced in the wild type under copper-depleted conditions, whereas production was markedly reduced upon copper supplementation (Fig. 4A to D and Table 2), thus supporting the expression profile of the two synthases (Fig. 2). However, minute levels of compounds 7 to 10 could still be detected under copper-replete conditions (Fig. 4A to D and Table 2). Given that loss of XanB alone is sufficient to abrogate production of all four xanthocillin derivatives, we conclude that the ICS5G BGC encodes xanthocillin biosynthetic pathway in A. fumigatus. As there are no predicted transporters within the *xan* BGC, and given that both *crmC* and *crmD* are upregulated during copper starvation (Fig. 2), we created a strain deleted for both transporters (*ΔcrmC ΔcrmD*) to assess whether these two putative metal transporters affect production of isocyanide derivatives in A. fumigatus (see Fig. S5 and Table S3 in the supplemental material). Despite being regulated in a copper-dependent manner, xanthocillin production in the *ΔcrmC ΔcrmD* mutant is similar to that of the wild-type strain grown under the same conditions (Fig. 4A to D and Table 2).

10.1128/mBio.00785-18.9 TABLE S3  Fungal strains used in this study. (See references 29 and 54 to 56 for details.) Download TABLE S3, DOCX file, 0.1 MB. Copyright © 2018 Lim et al. 2018 Lim et al. This content is distributed under the terms of the Creative Commons Attribution 4.0 International license.

**TABLE 2:** Production of xanthocillin derivatives in liquid shake culture (compounds 7 to 12), comparing the OE::*xanC* mutant to deletion strains

Compound	Production of derivative in^a^:							
	OE::*xanC* mutant		WT		Deletion mutant			
	−Cu	+Cu	−Cu	+Cu	Δ*crmA*	Δ*crmCD*	Δ*xanB*	Δ*xanB* Δ*crmA*
7	4.4E8 ± 2.1E8	1.7E9 ± 1.4E8	5.5E6 ± 2.2E6	1.9E6 ± 9.1E5	6.1E6 ± 2.5E6	1.8E6 ± 1.0E6	0	0
8	2.6E9 ± 8.7E8	4.6E9 ± 5.1E8	4.9E7 ± 9.0E6	5.1E6 ± 2.1E6	5.3E7 ± 2.8E7	5.9E7 ± 8.3E6	0	0
9	1.3E9 ± 4.3E8	1.3E9 ± 9.9E7	2.0E7 ± 1.8E6	1.1E6 ± 4.6E5	1.9E7 ± 9.7E6	2.9E7 ± 3.6E6	0	0
10	4.6E8 ± 2.2E8	4.3E9 ± 3.9E8	5.6E6 ± 1.3E6	1.5E6 ± 5.5E5	2.7E6 ± 1.4E6	1.4E6 ± 4.0E5	0	0
11	0	8.4E7 ± 2.6E7	0	0	0	0	0	0
12	0	9.9E6 ± 2.7E6	0	0	0	0	0	0

a The numbers indicate the average ± standard deviation for the peak intensity of each compound, as determined by LC-HRMS with four replicates. All deletion mutants were grown under copper-depleted conditions.

**FIG 5:**
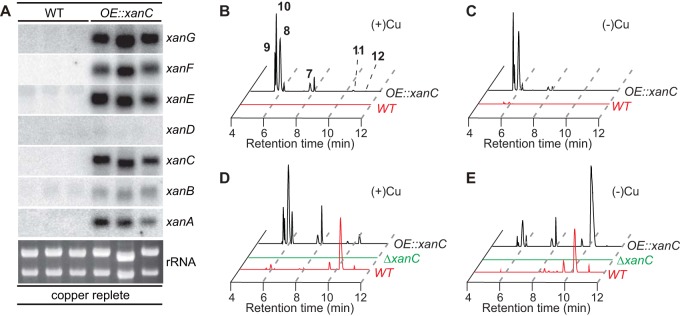
*xanC* regulates *xan* cluster gene expression and metabolite production. (A) Northern analysis depicting expression of *xanA* to -*G* in the both the wild type and OE::*xanC* mutant grown under copper-replete conditions. (B and C) Extracted-ion chromatograms (EICs) corresponding to compounds 7 to 12 in the wild type grown with or without copper (red lines) and the OE::*xanC* mutant with or without copper (black lines) in liquid shake culture. (D and E) EICs corresponding to compounds 7 to 12 in the wild type grown with or without copper (red lines), the OE::*xanC* mutant with or without copper (black lines), and the Δ*xanC* mutant (green lines) in a solid plate culture.

To further investigate regulation of the *xan* BGC, we overexpressed and deleted the sole transcription factor present in this cluster (XanC) to determine whether it controls expression of other cluster genes and formation of *xan* biosynthetic products (Fig. 1, Table S3, and Fig. S5). Supporting regulation of the *xan* BGC by XanC, expression of all *xan* genes (except *xanD*, encoding a putative protein with an uncharacterized domain [Fig. 1]) is upregulated in the OE::*xanC* mutant (Fig. 5A). LC-HRMS analysis of the XanC overexpression mutant (OE::*xanC*) revealed increased abundance of all xanthocillin derivatives compared to the wild type under both copper-replete (5 µM Cu) and -depleted conditions (Fig. 5B and C and Table 2). Further, analysis of OE::*xanC* revealed two additional isonitrile-containing compounds, the sulfated xanthocillin derivative BU-4704 (compound 11) (Fig. 4E, Table 1, Fig. S6D, and Table S2D) (18) and xanthocillin X monomethyl ether (compound 12) (Fig. 4F and Table 1). Table 2 compares the production of compounds 7 to 12 in the OE::*xanC* mutant to those of the other mutants grown in copper-depleted medium, showing much higher production of all metabolites in the overexpression strain, with the exception of compound 12, the nonsulfated xanthocillin X monomethyl ether. Table 2 also validates the results from Fig. 4, showing the requirement of *xanB* for compounds 7 to 12. In contrast, deletion of *xanC* virtually eliminates all pathway metabolites (Fig. 5D and E, Table 3, and Fig. S5).

Total production of *xan* biosynthetic products is elevated under all growth conditions in both the wild-type strain and OE::*xanC* mutant when A. fumigatus is grown on solid culture (Fig. 5D and E and Table 3). In-depth analysis of all intermediates showed that under liquid-submerged conditions, the production of dipeptides melanocins E (compound 9) and F (compound 10) is favored, whereas under solid-culture conditions, the isocyanide (compounds 11 and 12) and formamide (compound 8) moieties are favorably produced (Fig. 5B to E and Tables 2 and 3). The accumulation of isocyanide moieties from the *xan* biosynthetic pathway may provide the fungus with ecological benefits during asexual sporulation.

**TABLE 3:** Production of xanthocillin derivatives in solid plate culture (compounds 7 to 12), comparing the OE::*xanC* mutant strain to the *ΔxanC* deletion strain

Compound	Production of derivative in^a^:					
	WT		OE::*xanC* mutant		*ΔxanC* deletion mutant	
	+Cu	−Cu	+Cu	−Cu	+Cu	−Cu
7	6.6E7 ± 2.4E7	1.9E8 ± 4.1E7	1.6E9 ± 3.1E8	9.9E8 ± 5.7E8	0	0
8	2.3E7 ± 1.0E7	6.4E8 ± 2.2E8	3.8E9 ± 8.1E8	8.2E9 ± 1.7E9	0	0
9	2.8E5 ± 1.9E5	9.1E6 ± 6.2E6	1.3E7 ± 4.9E6	3.8E8 ± 2.5E8	0	1.1E5 ± 6.9E4
10	3.7E7 ± 8.2E6	4.8E7 ± 1.1E7	4.0E8 ± 2.4E8	5.9E8 ± 3.1E8	0	0
11	5.9E9 ± 7.3E8	6.4E9 ± 1.2E9	1.2E10 ± 2.3E9	6.3E8 ± 1.1E8	0	0
12	2.8E8 ± 4.8E7	1.6E8 ± 3.5E7	6.3E8 ± 1.1E8	3.9E7 ± 4.7E6	0	0

a The numbers indicate the average ± standard deviation for the peak intensity of each compound determined by LC-HRMS with three replicates.

### A model for xanthocillin biosynthesis in Aspergillus fumigatus.

**FIG 6:**
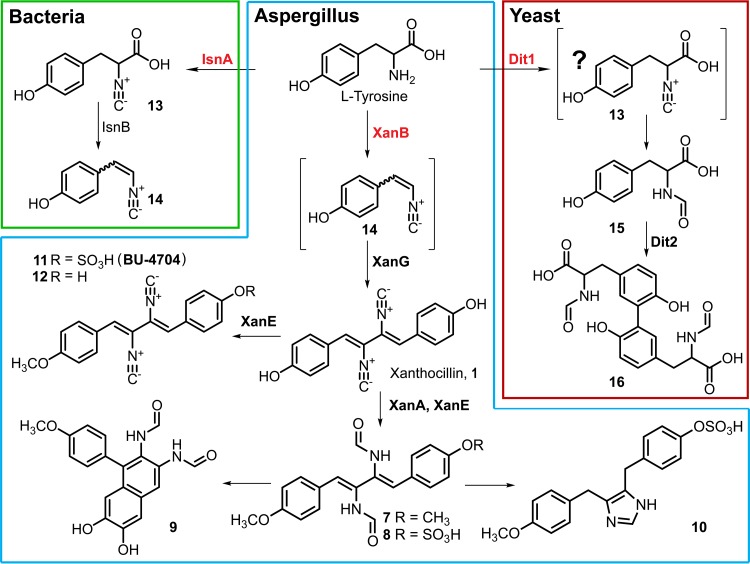
Putative biosynthesis of xanthocillin derivatives in A. fumigatus and related pathways in the bacterium Xenorhabdus nematophila (5) and yeast Saccharomyces cerevisiae (36). Tyrosine is converted into intermediate compound 14 by XanB, which is then converted by XanG into xanthocillin. *N*-Formyl and methyl moieties in xanthocillin derivatives are introduced by XanA and XanE, respectively. Fumiformamide (compound 8) is converted into melanocins E (compound 9**)** and F (compound 10**)** by oxidative and reductive cyclization, respectively. In yeast, tyrosine is converted into *N*-formyl tyrosine (compound 15) by Dit1, followed by dimerization via Dit2 to form *N*,*N*-bisformyl dityrosine (compound 16). The presence of isocyanide (compound 13**)** in yeast has not been established.

In conjunction with previous work on related enzymes in bacteria and yeast, our results suggest that XanB converts l-tyrosine to putative intermediate compound 14 in a two-step sequence analogous to bacterial IsnA-IsnB isocyanide synthase (Fig. 6) (5). Subsequent oxidative dimerization of compound 14 to form xanthocillin may involve XanG, a cytochrome P450 whose expression is coregulated with that of XanB. XanG is a homologue of the yeast enzyme Dit2, which catalyzes formation of *N*,*N*-bisformyl dityrosine, a spore wall component, which, similar to xanthocillin, is derived from dimerization of two tyrosine-derived moieties (Fig. 6) (36). As pointed out above, Dit1, the enzyme involved in the initial step of the yeast pathway to *N*,*N*-bisformyl dityrosine, shows high homology to other fungal ICSs and specifically the ICS domain of XanB, suggesting that the xanthocillin and *N*,*N*-bisformyl dityrosine biosynthetic pathways are closely related. The *N*-formyl moiety in compounds 7 to 9 is presumably introduced by XanA, a close homologue of a known isocyanide hydratase, which may act as part of isocyanide detoxification (37). The methyl moiety in six xanthocillin derivatives is likely introduced by the putative methyltransferase XanE. Finally, both melanocins E (compound 9) and F (compound 10) appear to be derived from the cyclization of compound 8 (Fig. 6).

## DISCUSSION

Many naturally occurring isocyanides have bioactivities of ecologic, economical, and pharmaceutical importance due to their structurally diverse carbon scaffolds and a highly reactive isocyanide moiety capable of coordinating transition metals. Despite their diverse biological activities, isocyanide biosynthesis had previously been studied only in bacteria. Our investigation of ICSs in the opportunistic human fungal pathogen A. fumigatus revealed the fungal xanthocillin biosynthetic pathway and hints at diverse functions of ICS homologues in eukaryotes.

**FIG 7:**
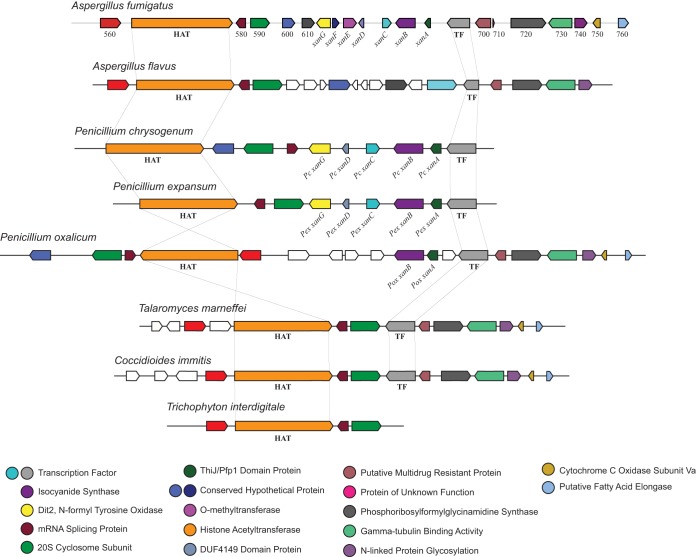
Comparison of the A. fumigatus *xan* BGC with *xan*-like BGCs in various other fungi. Genes showing similar homology are shaded with an identical color and listed with their corresponding function. Genes that do not show homology to genes found in the *xan* BGC are shown in white.

Based on the phylogeny of the ICS domain (PF05010), we found that A. fumigatus harbors all three variations of ICS domain proteins (Fig. 1 and 2). ICS homologues appear to be widely distributed in both bacteria and fungi, with outlier representation in oomycetes, plants, and metazoans (Fig. 2). Fungal ICSs do not appear to have been recently horizontally acquired from bacteria, are evolutionarily arranged into several distinct fungal clades, and occur particularly concentrated within the phylum Ascomycota (Fig. 2). Significantly, our examination of the ICS phylogeny revealed a recent evolutionary expansion of the novel ICS-NRPS hybrid enzymes in many important fungal pathogens and highlighted the presence of ICS homologues in a yeast taxon (Saccharomycotina) not normally known to harbor extensive secondary metabolism capabilities, including the human-pathogenic *Candida* spp. (Fig. 2A, Table S1, and Fig. S3). A detailed examination of the *xan* gene cluster architecture showed conservation of five *xan* genes (*xanA*, *xanB*, *xanC*, *xanD*, and *xanG*) in both Penicillium chrysogenum and Penicillium expansum (Fig. 7). P. expansum has been reported to produce the isocyanide xanthocillin X (38). However, only *xanA* and *xanB* are conserved in Penicillium oxalicum (Fig. 7). Conservation of flanking genes but not the *xan* BGC in various fungal genera extending to Talaromyces marneffei, Trichophyton interdigitale, and Coccidioides immitis suggests an insertion of the *xan* BGC in the Eurotiales (Fig. 7).

Yeast ICS homologues represent a family of proteins named Dit1, which are involved in the production of the sexual spore wall component *N*,*N*-bisformyl dityrosine in S. cerevisiae (27) and likely in C. albicans as well (39, 40). However, the specific enzymatic function of this protein, including its enzymatic product, remains unknown. In both S. cerevisiae and C. albicans, the *dit1* gene is transcribed divergently from *dit2*, encoding a cytochrome P450 monooxygenase, which is assumed to catalyze the formation of *N*,*N*-bisformyl dityrosine from the product of Dit1 catalysis (Fig. 6) (27). Dit1 and Dit2 are homologues of XanB and XanG in A. fumigatus, respectively. Given that the putative mechanism of *N*,*N*-bisformyl dityrosine formation is strikingly similar to that of xanthocillin biosynthesis, we propose the unknown product of Dit1 catalysis in S. cerevisiae to be a tyrosine isocyanide precursor (compound 13) (Fig. 6).

Our results demonstrate that in A. fumigatus, XanB is the dedicated synthase for production of the copper-responsive isocyanides and their derivatives, and we suggest that the Dit2-like protein XanG catalyzes oxidative dimerization, following isocyanide formation via XanB. We showed that both copper-fist transcription factors (AceA and MacA) are involved in governing the copper-responsive profile of the *xan* and *crm* BGCs. Although the *crm* BGC is similarly regulated by copper availability, we find no direct involvement of this BGC in the production of compounds 7 to 12. Given that CrmA is a novel type of ICS-NRPS hybrid enzyme within the ICS3G-BGC found to be enriched in many fungal pathogens of plants, insects, and humans, our ongoing work aims to elucidate the activity of this enzyme and identify its biosynthetic product(s), to further define the copper-responsive regulation of its gene cluster, and ultimately to query their role or roles in the ecologic fitness or pathogenicity potential of A. fumigatus and other pathogenic fungi. The involvement of this family of ICSs in fungi, on one hand, to produce an important cell wall dipeptide (dityrosine) in yeast, and on the other hand, to produce a potential defensive molecule (xanthocillin) in filamentous mold makes for their interesting position at the interface of both primary and secondary metabolism.

The discovery of diverse ICSs in fungi revealed a novel, unexplored aspect of fungal secondary metabolism. Whereas iron-responsive secondary metabolism in fungi is well understood, our characterization of xanthocillin biosynthesis and regulation of the synthases involved in its production in A. fumigatus offers the first insight into copper-responsive secondary metabolism in fungi, possibly related to copper chelation processes, as has been found for a few other natural products, such as yersiniabactin and methanobactins (41, 42). Finally, it should be noted that two of the identified ICS BGCs were “invisible” to current secondary metabolite prediction algorithms, including antiSMASH (26) and SMURF (25) because of the lack of precedent for eukaryotic ICSs and absence of canonical class-defining secondary-metabolite-producing synthases (e.g., nonribosomal peptide synthetase, polyketide synthase, terpene cyclase, prenyltransferase, etc.). Revision of such algorithms will further expand the power of bioinformatically driven natural product mining.

## MATERIALS AND METHODS

### Fungal growth and culture conditions.

All A. fumigatus strains used in this study are listed in Table S3. Strains were maintained as glycerol stocks and activated on solid glucose minimal medium (GMM) at 37°C (37). Growth medium was supplemented with 5.0 mM uridine and uracil and 5.7 mM arginine for *pyrG* and *argB* auxotrophs, respectively. For isolation of genomic DNA (gDNA) for PCR and Southern blotting, 10 ml of liquid minimal medium (43) with yeast extract was inoculated with spores from solid medium and grown overnight at 37°C. For isolation of the OE::*xanC* and wild-type AF293 RNA, 50 ml of GMM was inoculated (1.0 × 10^6^ spores per ml), and cells were grown for ~12 h at 37°C with shaking at 200 rpm. For cDNA synthesis of *crmA*, A. fumigatus AF293 was inoculated at 1.0 × 10^6^ spores per ml into yeast extract-glucose (YG) medium for the inducing condition and YG medium plus trace elements for the noninducing condition and cultured at 37°C for 24 h. For ultrahigh-performance liquid chromatography (UHPLC)-HRMS and NMR analysis, strains were inoculated (1.0 × 10^6^ spores per ml) into 50 ml GMM with (5 µM) and without copper supplementation in a 125-ml Erlenmeyer flask at 37°C with shaking at 200 rpm for 120 h. For metabolite analysis under solid conditions, 1 × 10^8^ total spores were plated onto GMM solid plates with (5 µM) and without copper. For copper-starved growth, conidia used for inoculation were harvested from copper-depleted GMM. For copper-replete growth, conidia used for inoculation were harvested from GMM containing 5 µM supplemented copper.

### Mutant construction.

The mutants used in this study (Table S3) were created using the double-joint PCR (DJ-PCR) method (44). Genomic DNA and RNA were isolated from A. fumigatus AF293 using standard procedures. Construction of DJ-PCR products, protoplast production, and transformation were carried out as previously described (44). Briefly, primers were designed to amplify approximately 1,000-bp flanks with 20 bp of overlap of the selection marker-containing plasmid using SeqBuilder within the Lasergene12 suite (DNASTAR, Madison, WI). For deletion strains, plasmid pJW24 (45) or pJMP4 (46) was used to amplify the A. parasiticus *pyrG* or A. fumigatus *argB* genes, respectively. For the *xanC* overexpression strain (OE::*xanC* mutant), plasmid pJMP9.1 (47) was used to amplify the A. parasiticus *pyrG* gene fused to an A. nidulans *gpdA* constitutively active promoter. Both *pyrG*^*−*^ (TFYL80) and *argB*^*−*^ (TFYL84) mutants were generated by complementing the *pyrG-argB*^*−*^ double auxotroph (TFYL45) with A. fumigatus *argB* (pJMP4) and A. fumigatus *pyrG* (pKJA12), respectively. The *ΔcrmA pyrG*^*−*^ mutant (TFYL90) was generated from TFYL45. The *ΔcrmA* mutant (TFYL93) was generated from TFYL84. The *ΔxanB* mutant (TFYL105) was generated from TFYL80.1. The *ΔcrmA ΔxanB* double mutant was generated from TFYL90. The OE::*xanC* mutant was generated from TFYL80. Transformants were screened for proper integration of the construct and loss of the gene of interest via PCR. Single integration of the transformation cassette was verified by Southern analysis (Fig. S5). Expression of *xanC* in the OE::*xanC* mutant was confirmed via Northern analysis. For all auxotrophic mutants, maintenance of the mutant allele(s) was confirmed via PCR after complementation to prototrophy.

### cDNA synthesis and cloning of *crmA*.

Fungal mycelia were harvested from cells under both inducing and noninducing conditions and lyophilized. Total RNA was extracted from the lyophilized mycelia using TRIzol according to the manufacturer’s protocol. Ten micrograms of total RNA was digested with DNase I and subjected to cDNA synthesis using the SuperScript III first-strand reverse transcriptase system according to the manufacturer’s protocol. The full-length *crmA* genes according to the new prediction (Fig. S1) were amplified and cloned into bacterial expression vector pTEV5 (48) to give rise to pFYL15.

### Phylogenetic analysis.

To identify isocyanide synthases in A. fumigatus, a PSI-BLAST search using amino acid sequences of the bacterial isocyanide synthase from P. aeruginosa, PvcA, was queried against the genome of A. fumigatus AF293. To build the phylogenetic trees, the regions corresponding to isocyanide synthase (Pfam domain PF05141), Fe^2+^ α-ketoglutarate-dependent dioxygenase (Pfam domain PF02668) in the ICSs of Aspergillus fumigatus AF293, CrmA, and XanB, were queried against Ensembl Genomes using phmmer, a member of the HMMER3 software suite (49) (web server accessed 15 July 2016). Significant hits were downloaded and filtered based on length and sequence composition and to reduce redundancy in the data set using a custom perl script as well as IQ-TREE (50) (Table S1). Following this filtering step, regions corresponding to each domain of interest were aligned with MAFFT v7.023b using the G-INS-I strategy (51), and the topologies were inferred using maximum likelihood (ML) as implemented in RAxML version 8.0.25 (52) using a PROTGAMMALG substitution model (automatically determined to be the best model within RAxML) and rapid bootstrapping (100 replications). All alignments and trees are available for download on the figshare data repository (https://doi.org/10.6084/m9.figshare.4721116.v1). The phmmer-identified sequences were scanned for significant hits to additional Pfam domains using an hmmsearch *E* value inclusion threshold of 0.001 (Table S1).

### Analytic methods and equipment overview. (i) NMR spectroscopy.

NMR spectroscopy was performed on a Bruker Avance III HD (800-MHz ^1^H reference frequency, 201 MHz for ^13^C) equipped with a 5-mm CPTCL ^1^H-^13^C/^15^N cryo probe. Non-gradient phase-cycled double quantum-filtered correlation spectroscopy (DQF-COSY) spectra were acquired using the following parameters: 0.6-s acquisition time; 400 to 600 complex increments, and 8, 16, or 32 scans per increment. Heteronuclear single quantum coherence (HSQC) and heteronuclear multiple bond correlation (HMBC) spectra were acquired with the following parameters: 0.25-s acquisition time, 200 to 500 complex increments, and 8 to 64 scans per increment. ^1^H,^13^C-HMBC spectra were optimized for *J*_H,C_ = 6 Hz. HSQC spectra were acquired with or without decoupling. NMR spectra were processed and baseline corrected using MestreLabs MNOVA software packages.

### (ii) MS.

Ultrahigh-performance liquid chromatography-high-resolution MS (UHPLC-HRMS) was performed on a Thermo Scientific-Dionex Ultimate 3000 UHPLC system equipped with a diode array detector and connected to a Thermo Scientific Q Exactive Orbitrap operated in electrospray ionization-positive (ESI^+^) or -negative (ESI^−^) mode.

### (iii) Chromatography.

Semipreparative chromatography was performed on an Agilent 1100 series HPLC system using an Agilent Zorbax Eclipse XDB-C_8_ column (25 cm by 10 mm, 5-µm particle diameter).

### Metabolite extraction and LC-MS analysis.

Liquid fungal cultures (50 ml) including fungal tissue and medium were frozen using a dry ice-acetone bath and lyophilized. The lyophilized residues were extracted with 20 ml of ethyl acetate-methanol (9:1) for 1.5 h with vigorous stirring. Extracts were filtered over cotton, evaporated to dryness, and stored in 4-ml vials. Crude extracts were suspended in 0.5 ml of methanol and centrifuged to remove insoluble materials, and the supernatant was subjected to UHPLC-HRMS analysis. An Agilent Zorbax RRHD Eclipse XDB-C_18_ column (2.1 by 100 mm, 1.8-µm particle diameter) was used with acetonitrile (organic phase) and 0.1% formic acid in water (aqueous phase) as solvents at a flow rate of 0.5 ml/min. A solvent gradient scheme was used, starting at 2% organic for 1 min, followed by a linear increase to 100% organic over 14 min, holding at 100% organic for 2.5 min, decreasing back to 2% organic for 0.1 min, and holding at 2% organic for the final 1.4 min, for a total of 18 min.

### Chromatographic purification of compounds 7, 9, 10, and 11.

Liquid fungal cultures (1 liter) including fungal tissue and medium were frozen using a dry ice-acetone bath and lyophilized. The combined lyophilized residues were extracted with 500 ml of ethyl acetate-methanol (9:1) for 3.5 h with vigorous stirring. Extracts were filtered over cotton, evaporated to dryness, and stored in 8-ml vials. Crude extracts were fractionated via semipreparative HPLC using an Agilent XDB C_8_ column (25 cm by 10 mm, 5-µm particle diameter) with acetonitrile (organic phase) and 0.1% acetic acid in water (aqueous phase) as solvents at a flow rate of 3.2 ml/min. A solvent gradient scheme was used, starting at 5% organic for 3 min, followed by a linear increase to 100% organic over 27 min, holding at 100% organic for 5 min, decreasing back to 5% organic for 0.1 min, and holding at 5% organic for the final 4.9 min, for a total of 40 min. Further purification of fractions containing compounds 7, 9, 10, and 11 was accomplished by semipreparative HPLC using an Agilent XDB C_8_ column (25 cm by 10 mm, 5-µm particle diameter) with acetonitrile (organic phase) and 0.1 M (pH 8.0) ammonium acetate in water (aqueous phase) as solvents at a flow rate of 3.2 ml/min with same gradient scheme described above.
