# Supplementary figures and images for: Fungal Isocyanide Synthases and Xanthocillin Biosynthesis in Aspergillus fumigatus
Source: mBio. 2018 May 29;9(3):e00785-18. doi: 10.1128/mBio.00785-18 (PMC5974471; doi:10.1128/mBio.00785-18)

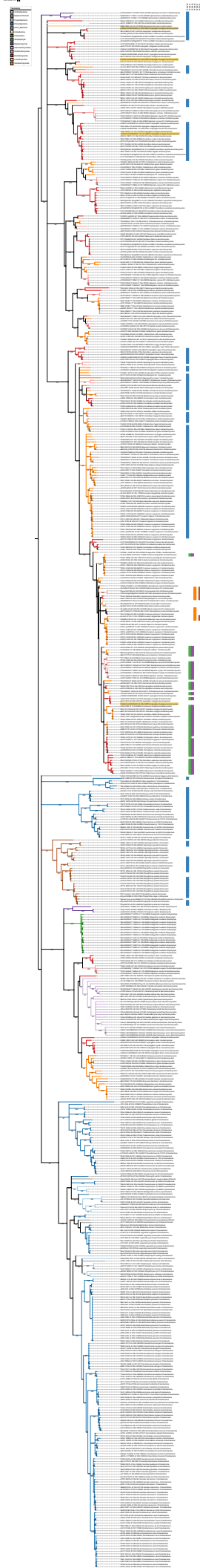

Supplement: FIG S3 [file mbo003183905sf3.pdf]

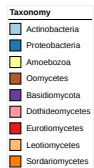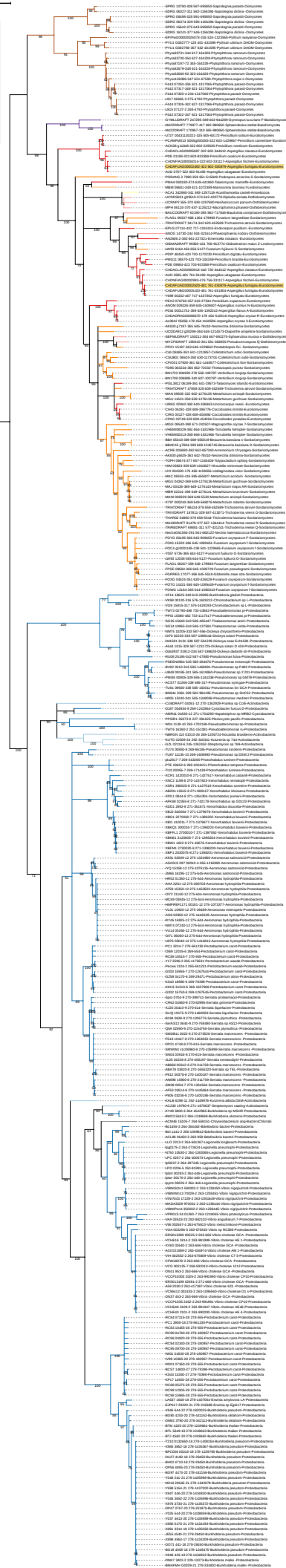[illegible]

Supplement: FIG S4 [file mbo003183905sf4.pdf]
