# Supplementary material for: Fungal Isocyanide Synthases and Xanthocillin Biosynthesis in Aspergillus fumigatus
Source: mBio. 2018 May 29;9(3):e00785-18. doi: 10.1128/mBio.00785-18 (PMC5974471; doi:10.1128/mBio.00785-18)
Supplement: TABLE S2 [file mbo003183905st2.docx]

**Table S2A.** ^1^H (800 MHz) and ^13^C (201 MHz) NMR spectroscopic data for *N*,*N'*-((1Z,3Z)-1,4-bis(4-methoxyphenyl)buta-1,3-diene-2,3-diyl)diformamide (**7**) in DMSO-*d*_6_.

Chemical shifts were referenced to δ(CHD_2_SOCD_3_) = 2.50 and δ(^13^CHD_2_SOCD_3_) = 39.5. ^13^C chemical shifts were determined via HMBC and HSQC spectra. ^1^H, ^1^H-*J*-coupling constants were determined from the acquired ^1^H or dqfCOSY spectra. HMBC correlations are from the proton(s) stated to the indicated ^13^C atom.

| **No.** | δ**c** | **Proton** | δ**H (*J*_HH_[Hz])** | **HMBC** |
| --- | --- | --- | --- | --- |
| 1 | 121.9, 122.9 | 1-H | 6.53, 6.55 | 2, 2', 6' |
| 2 | 130.0, 130.5 |  |  |  |
| 3 | 131.3, 131.6 |  |  |  |
| 4 | 120.1, 121.5 | 4-H | 6.50, 6.55 | 2, 2'', 6'' |
| 5 |  | 5-H | 9.30 (*J*_5,6_ = 8.6), 9.37 (*J*_5,6_ = 8.6) | 2, 3, 4, 6 |
| 6 | 164.1 | 6-H | 7.79 (*J*_5,6_ = 8.6), 7.86 (*J*_5,6_ = 8.6) | 3 |
| 7 |  | 7-H | 9.46, 9.56 | 1, 2, 3, 8 |
| 8 | 159.9 | 8-H | 8.18, 8.19 | 2 |
| 1' | 127.6, 127.9 |  |  |  |
| 2' | 130.0, 130.1 | 2'-H | 7.45 (*J*_2',3'_ = 8.6), 7.47 (*J*_2',3'_ = 8.6) | 1, 4' |
| 3' | 113.5, 113.6 | 3'-H | 6.91 (*J*_3',2'_ = 8.6), 6.93 (*J*_3',2'_ = 8.6) | 1' |
| 4' | 158.4, 158.7 |  |  |  |
| 5' | 113.5, 113.6 | 5'-H | 6.91 (*J*_3',2'_ = 8.6), 6.93 (*J*_3',2'_ = 8.6) | 1' |
| 6' | 130.0, 130.1 | 6'-H | 7.45 (*J*_2',3'_ = 8.6), 7.47 (*J*_2',3'_ = 8.6) | 1, 4' |
| 7' | 54.8 | 7'-H | 3.77 | 4' |
| 1'' | 127.2, 127.6 |  |  |  |
| 2'' | 130.2, 130.3 | 2''-H | 7.42 (*J*_2',3'_ = 8.6), 7.44 (*J*_2',3'_ = 8.6) | 1, 4' |
| 3'' | 113.6, 113.8 | 3''-H | 6.93 (*J*_3',2'_ = 8.6), 6.95 (*J*_3',2'_ = 8.6) | 1' |
| 4'' | 158.2, 158.4 |  |  |  |
| 5'' | 113.6, 113.8 | 5''-H | 6.93 (*J*_3',2'_ = 8.6), 6.95 (*J*_3',2'_ = 8.6) | 1' |
| 6'' | 130.2, 130.3 | 6''-H | 7.42 (*J*_2',3'_ = 8.6), 7.44 (*J*_2',3'_ = 8.6) | 1, 4' |
| 7'' | 54.8 | 7''-H | 3.77 | 4' |

**Table S2B.** ^1^H (800 MHz) and ^13^C (201 MHz) NMR spectroscopic data for melanocin E (**9**) in methanol-*d*_4_.

Chemical shifts were referenced to δ(CHD_2_OD) = 3.31 and δ(^13^CHD_2_OD) = 49.0. ^13^C chemical shifts were determined via HMBC and HSQC spectra. ^1^H, ^1^H-*J*-coupling constants were determined from the acquired ^1^H or dqfCOSY spectra. HMBC correlations are from the proton(s) stated to the indicated ^13^C atom.

| **No.** | δ**c** | **Proton** | δ**H (*J*_HH_[Hz])** | **HMBC** |
| --- | --- | --- | --- | --- |
| 1 | 137.4 |  |  |  |
| 2 | 122.4 |  |  |  |
| 3 | 129.9 |  |  |  |
| 4 | 119.3 | 4-H | 8.30 | 2, 5, 8a |
| 4a | 130.0 |  |  |  |
| 5 | 110.2 | 5-H | 7.12 | 4, 7, 8a |
| 6 | 148.3 |  |  |  |
| 7 | 147.4 |  |  |  |
| 8 | 109.4 | 8-H | 6.72 | 1, 4a, 6 |
| 8a | 127.6 |  |  |  |
| 9 | 163.1 | 9-H | 8.01 | 2 |
| 10 | 161.9 | 10-H | 8.33 | 3 |
| 1' | 130.3 |  |  |  |
| 2' | 131.8 | 2'-H | 7.18 (*J*_2',3'_ = 8.6) | 1, 4' |
| 3' | 114.5 | 3'-H | 7.04 (*J*_3',2'_ = 8.6) | 1' |
| 4' | 160.4 |  |  |  |
| 5' | 114.5 | 5'-H | 7.04 (*J*_5',6'_ = 8.6) | 1' |
| 6' | 131.8 | 6'-H | 7.18 (*J*_6',5'_ = 8.6) | 1, 4' |
| 7' | 55.5 |  | 3.86 | 4' |

**Table S2C.** ^1^H (800 MHz) and ^13^C (201 MHz) NMR spectroscopic data for melanocin F (**10**) in methanol-*d*_4_.

Chemical shifts were referenced to δ(CHD_2_OD) = 3.31 and δ(^13^CHD_2_OD) = 49.0. ^13^C chemical shifts were determined via HMBC and HSQC spectra. ^1^H, ^1^H-*J*-coupling constants were determined from the acquired ^1^H or dqfCOSY spectra. HMBC correlations are from the proton(s) stated to the indicated ^13^C atom.

| **No.** | δ**c** | **Proton** | δ**H (*J*_HH_[Hz])** | **HMBC** |
| --- | --- | --- | --- | --- |
| 1 | 29.2 | 1-H | 4.03 | 2, 3, 1', 2', 6' |
| 2 | 130.2 |  |  |  |
| 3 | 129.5 |  |  |  |
| 4 | 29.3 | 4-H | 4.04 | 2, 3, 1'', 2'', 6'' |
| 5 | 133.6 | 5-H | 8.70 | 2, 3 |
| 1' | 129.7 |  |  |  |
| 2' | 130.2 | 2'-H | 7.08 (*J*_2',3'_ = 8.7) | 1, 4' |
| 3' | 115.1 | 3'-H | 6.87 (*J*_3',2'_ = 8.7) | 1' |
| 4' | 160.0 |  |  |  |
| 5' | 115.1 | 5'-H | 6.87 (*J*_5',6'_ = 8.7) | 1' |
| 6' | 130.2 | 6'-H | 7.08 (*J*_6',5'_ = 8.7) | 1, 4' |
| 7' | 55.4 | 7'-H | 3.77 | 4' |
| 1'' | 134.2 |  |  |  |
| 2'' | 129.8 | 2'-H | 7.10 (*J*_2',3'_ = 8.7) | 4, 4'' |
| 3'' | 122.7 | 3'-H | 7.23 (*J*_3',2'_ = 8.7) | 1'' |
| 4'' | 152.8 |  |  |  |
| 5'' | 122.7 | 5'-H | 7.23 (*J*_3',2'_ = 8.7) | 1'' |
| 6'' | 129.8 | 6’-H | 7.10 (*J*_2',3'_ = 8.7) | 4, 4'' |

**Table S2D.** ^1^H (800 MHz) and ^13^C (201 MHz) NMR spectroscopic data for BU-4704 (**11**) in methanol-*d*_4_. Chemical shifts were referenced to δ(CHD_2_OD) = 3.31 and δ(^13^CHD_2_OD) = 49.0. ^13^C chemical shifts were determined via HMBC and HSQC spectra. ^1^H, ^1^H-*J*-coupling constants were determined from the acquired ^1^H or dqfCOSY spectra. HMBC correlations are from the proton(s) stated to the indicated ^13^C atom.

| **No.** | δ**c** | **Proton** | δ**H (*J*_HH_[Hz])** | **HMBC** |
| --- | --- | --- | --- | --- |
| 1 | 128.2 | 1-H | 7.14 | 2, 2'', 3 |
| 2 | 117.0 |  |  |  |
| 3 | 118.7 |  |  |  |
| 4 | 129.2 | 4-H | 7.13 | 6'' |
| 5 | ND |  |  |  |
| 6 | ND |  |  |  |
| 1' | 125.8 |  |  |  |
| 2' | 132.8 | 2'-H | 7.85 (*J*_2',3'_ = 8.7) | 1, 4' |
| 3' | 115.3 | 3'-H | 7.06 (*J*_3',2'_ = 8.7) | 1' |
| 4' | 162.7 |  |  |  |
| 5' | 115.3 | 5'-H | 7.06 (*J*_5',6'_ = 8.7) | 1' |
| 6' | 132.8 | 6'-H | 7.85 (*J*_6',5'_ = 8.7) | 1, 4' |
| 7' | 55.7 | 7'-H | 3.87 | 4' |
| 1'' | 129.6 |  |  |  |
| 2'' | 131.9 | 2''-H | 7.84 (*J*_2'',3''_ = 8.5) | 4'', 4 |
| 3'' | 122.2 | 3''-H | 7.43 (*J*_3'',2''_ = 8.5) | 1'' |
| 4'' | 155.3 |  |  |  |
| 5'' | 122.2 | 5''-H | 7.43 (*J*_5'',6''_ = 8.5) | 1'' |
| 6'' | 131.9 | 6''-H | 7.84 (*J*_6'',5''_ = 8.5) | 4'', 4 |
