# Supplementary material for: Fungal Isocyanide Synthases and Xanthocillin Biosynthesis in Aspergillus fumigatus
Source: mBio. 2018 May 29;9(3):e00785-18. doi: 10.1128/mBio.00785-18 (PMC5974471; doi:10.1128/mBio.00785-18)
Supplement: TABLE S3 [file mbo003183905st3.docx]

**Table S3.** Fungal strains used in this study.

| **Strain** | **Identifier** | **Genotype** | **Source** |
| --- | --- | --- | --- |
| AF293 | *WT* | *Wild type* | (54) |
| TFYL45 | *pyrG- argB-* | *∆nkuA::mluc; pyrG1; argB1* | (55) |
| TFYL81 | *WT* | *fumipyrG; fumiargB; ∆nkuA::mluc; pyrG1; argB1* | (55) |
| TFYL80 | *pyrG-* | *fumiargB; ∆nkuA::mluc; pyrG1; argB1* | This study |
| TFYL84 | *argB-* | *fumipyrG; ∆nkuA::mluc; pyrG1; argB1* | This study |
| TFYL89 | *∆crmC∆crmD* | *∆crmC∆crmD::fumiargB; fumipyrG; ∆nkuA::mluc; pyrG1; argB1* | This study |
| TFYL90 | *∆crmApyrG-* | *∆crmA::fumiargB; ∆nkuA::mluc; pyrG1; argB1* | This study |
| TFYL93 | *∆crmA* | *∆crmA::fumiargB; fumipyrG; ∆nkuA::mluc; pyrG1; argB1* | This study |
| TFYL105 | *∆xanB* | *∆xanB::parapyrG; fumiargB; ∆nkuA::mluc; pyrG1; argB1* | This study |
| TFYL106 | *∆crmA∆xanB* | *∆crmA::fumiargB; ∆xanB::parapyrG; ∆nkuA::mluc; pyrG1; argB1* | This study |
| TNLR 1 | *OE::xanC* | *parapyrG::gpdA(p)::xanC; fumiargB; ∆nkuA::mluc; pyrG1; argB1* | This study |
| TNLR 9 | *∆xanC* | *∆xanC::parapyrG; fumiargB; ∆nkuA::mluc; pyrG1; argB1* | This study |
| CEA17 KU80 | *WT* | *pyrG1*, ∆*akuB*::*pyrG* | (56) |
| *∆macA* | *∆macA* | *pyrG1,* ∆*akuB*::*pyrG*, *pyrG1*, ∆*AFUB_012670*::*pyrG* | (29) |
| *∆aceA* | *∆aceA* | *pyrG1,* ∆*akuB*::*pyrG*, *pyrG1*, ∆*AFUB_073740*::*pyrG* | (29) |
| *∆cufA* | *∆cufA* | *pyrG1,* ∆*akuB*::*pyrG*, *pyrG1*, ∆*AFUB_018270*::*pyrG* | (29) |
